# President’s Address

**Published:** 1873-04

**Authors:** G. W. Holmes

**Affiliations:** Rome, Georgia


### PRESIDENT'S ADDRESS,

*Delivered Before the Georgia Medical Association, Atlanta, Georgia, April 9, 1873.*

BY G. W. HOLMES, M.D., ROME, GEORGIA.

*Gentlemen of the Convention:*—At your last annual meeting, you did me the honor to elect me your presiding officer. This, in connection with the by-laws governing your body, imposes upon me the duty of delivering an address; which, gentlemen, affords me the opportunity of expressing my gratitude to the members of the Association, and of assuring them I feel a deep and abiding interest in each and every object for which it was formed, and confidently look forward to their perfect realization. Twenty-four years have been numbered in the past since its organization; many of its members have paid the inexorable demand of nature, and in our annual deliberations their voices can never again be heard, communicating the rich stores of practical wisdom, which lives of enthusiastic devotion to a noble profession had achieved. Let us who yet live know that the formation of the Georgia Medical Association has done more to elevate and dignify the medical profession of the State than all other means combined, and as we love and honor our profession so will we love, honor and cherish the memory of those who originated and kept alive so potent a lever for its elevation.

It has been no easy task to carry the Association to its present status. Its organic functions have been put to a fearful test, but, fortunately for its own preservation and the honor of the profession, it contained among its members wise, good and self-sacrificing men, who, Clay and Webster like, threw themselves in the breach, and, by the sacrifice of time and money, coupled with a devotion equal to the occasion, compromised the disturbing elements and reëstablished a strength and harmony made stronger and purer by the severe ordeal through which it had passed. Let us never, no never, cease to cherish, honor and love the noble, self-sacrificing spirit that seeks to protect and perpetuate the Georgia Medical Association, until it shall become so old that the pleasant reminiscences of the past shall cluster around its annual meetings with a hallowed influence, sufficient to perpetuate its existence as long as time shall last.

The formation of a Medical Association was freely discussed several years before its consummation, and it is not a little surprising, when we consider the importance of the enterprise, that it was so long postponed. Even now it can not be said it is all that we would have it. Notwithstanding the labors and well directed efforts of its members, much remains to be done to give it efficiency and to extend its usefulness. Yet we may congratulate ourselves upon having made a good beginning; and if we proceed in the proper spirit—the spirit of kindly feeling and cordial sympathy—earnestly carrying out our plans and intentions, it is easy to foresee what will be the happy result.

"In union there is strength," is an old maxim that has been acted upon by all men, of whatever pursuit, interest or occupation; and, I believe, in no age of the world has its practical force been more fully illustrated than the present—an age emphatically far in advance, in progress and improvement, of any that has preceded it.

Concentration of action will accomplish in a day what single-handed labor will not in a life-time. All great efforts are made successful by coöperation and concert of action. It is thus that nations achieve their independence, and triumph over tyranny and oppression; that learning and science, like the rays of the sun, penetrate the remotest corners of the earth.

At this meeting, let us resolve that henceforth we will be united in our efforts to be more faithful to our high vocation, that its dignity may be maintained and its usefulness extended. This determined and carried out in good faith will crown our efforts with honor to ourselves and benefit to our profession. When the time shall have arrived that each and every member of this Assocation shall fully determine in his own mind to do all in his power to elevate the standard of medical education, to improve the morals of the profession, and have its members feel the magnitude of the many responsibilities resting upon them, not only as medical gentlemen, but members of society; in a word, when we determine, individually and collectively, to do all we can to carry out that most excellent and unexceptionable code, erected by our predecessors as a ladder of easy ascension, at the summit of which may be found the high and noble attainments that belong to our time-honored profession; then may we confidently expect a rapid growth of our chosen science, of sufficient strength to throw off from its surface the many foul and disturbing excrescences that have retarded its growth, and see it ripen into a respectability that will be recognized and kept pure by the Government, as it is in the mother country.

It is unreasonable to expect to be respected or protected by the Government until we make ourselves respectable and worthy of protection. This end is certainly attainable, for there is no pursuit or avocation of man founded in truth and based upon the incontrovertible laws of nature, that can be fettered and crushed unto death. It may be retarded and sleep until a generation passes away; its admirers and devotees, perplexed and disappointed, may pass away, but only to give place to others who will press bravely onward, and bring it nearer to perfection. Thus it will go from generation to generation, until it becomes a living principle, acknowledged, respected and protected.

Let us look at the past history of our science, and see how it has steadily advanced, although at many times fettered and confined, but only to be loosened and advanced still farther, when, by its many virtues and acknowledged truths, it became self-protecting; or in other words, so respectable and meritorious that, to-day, in the very country where it was retarded by the Government it stands preëminently triumphant, with her strong arm thrown around it. If this be so, let us be encouraged and be true to our calling—making ourselves master-workmen for its advancement in America—and the day is not far distant when we shall have seen our labors crowned with like success.

Let us move steadily on, deeply impressed with the importance of investigating the hidden truths of the science, applying the good results to the benefit of mankind, keeping constantly in view the many responsibilities resting upon us, never forgetting, that by reason of our professional position, that it is largely in our power to give tone and impress of such a character to society as not only to prevent disease and elevate our race, but inure to the advancement of the profession we represent. Let us acquire knowledge based upon the immutable laws of nature, actively working in the wide field of science, and our influence will be felt beyond the limits of the sick-room: reacting favorably upon fame and fortune, diffusing its grateful and humanizing effects through all the social and civil relations of life.

It would be a pleasing task to speak of the many improvements in medicine during our epoch, but it would consume much valuable time, and be nothing new to you, my associates, as the changes have been of such striking importance as to elicit comment from even the non-professional. But I will suggest that we allow ourselves for a moment to think of the gigantic strides that have been and are still being made, that we may, at this our twenty-fourth annual meeting, be encouraged to renewed energies to contribute all in our means (be it ever so humble) to assist in adding something to the general fund of information already accumulated.

Now, my brethren, while in the "Gate City," which teems with joyous hope and busy enterprise that seems to leap to laughing life, let us be alike enthused to active work in furthering the objects of our present convocation. We have here assembled the representative talent and experience of our profession from all parts of the State, and while holding this annual medical lovefeast, let us talk freely one to another, giving our full experience, humble though it may be. It is thus we may be able to strengthen our opinions, confirm our experience, and elucidate facts that shed more light upon theories—facilitating the exploration of their many dark labyrinths, enabling the investigator to move rapidly onward, crowning our science with new and valuable discoveries.

In order that this very desirable object may be attained, I would most respectfully beg of you never to allow any other than a medical subject to come before the Association for disdussion. I am fully aware that there are many subjects in relation to ethics that may arise, and should have prompt and decisive action. To meet this requirement, I would respectfully suggest that a Board of Censors, consisting of five, be selected, to whom all questions of difference and doubt may be referred, making their decision final. This being done, it would be well understood that nothing would be taken under consideration by the body, except such subjects as appertain strictly to medicine. This would make our meetings of such vital interest that the progressive portion of the profession could not afford to be absent. We would then have more time for reports and debates —the only source within our power to arrive at the full sense of the meeting on the many opinions and theories that may be presented, which is the prime object of most members, and the very essence of the Association.

When the profession becomes fully alive to the importance of meeting annually, reporting and discussing the subjects of interest that may be presented from the entire State, our proceedings will be massive, and of great interest to the profession, and especially the members of the Association, making a volume that none who claim to be fully up with the times can afford to be without. This would necessarily impose much careful labor of a protracted nature upon the Secretary, and should be met by an appropriate salary, which would require but a trifle in the way of an assessment, when compared with the value of the fresh opinions of the profession in a neat and well arranged volume to its members, and I have no doubt but that it would meet the hearty approbation of every one.

I would further suggest the importance of procuring a State Board of Health, as the best means of enlightening the public mind, and of obtaining such legislative enactments as are absolutely necessary for the good of the public. When an intelligent people are made fully acquainted with the necessity of legislative action for their own preservation, it is reasonable to suppose they will not be long in giving it. The physician, as a "watchman on the tower," held responsible by an exacting public, should be armed with sufficient power to enable him to do his honest duty, and meet the stern demand of an enlightened people.

And now, my fellow-members, in conclusion let me say the profession we represent is as ancient as time, dating at least as far back as the time when man lost his primal perfection, and pain and suffering came into the world. It was regarded by the remotest nations as sacred, and worthy of the greatest respect and admiration. It was recognized and dispensed with a prodigal hand by the founder of Christianity, which should forever hallow and dignify it. Let us exert ourselves to maintain our ancient and modern position, and acquire wisdom and character, which are the essence of true education, and the light which will enable us to add new merit and perpetuate its reputation, until its great learning and many virtues shall rise, like the "god of day," and shine more and more unto meridian splendor.

